# The Effect of Lower Limb Pressotherapy Treatment on Selected Rheological and Biochemical Indices of Blood in Young, Healthy Women

**DOI:** 10.3390/jcm13195743

**Published:** 2024-09-26

**Authors:** Bartłomiej Ptaszek, Anna Wójciak, Angelika Żak, Szymon Podsiadło

**Affiliations:** 1 Institute of Applied Sciences, University of Physical Education in Krakow, 31-571 Krakow, Poland; 2 Faculty of Motor Rehabilitation, University of Physical Education in Krakow, 31-571 Krakow, Poland; 3 Institute of Clinical Rehabilitation, University of Physical Education in Krakow, 31-571 Krakow, Poland

**Keywords:** pressotherapy, blood rheology, lipid profile, renal profile

## Abstract

**Background:** Intermittent pneumatic compression is a non-invasive therapeutic technique that has been gaining popularity in recent years due to its potential use in many areas of medicine. It can be successfully used alone or in combination with other therapeutic methods. The aim of this study was to investigate whether and how a series of pressotherapy treatments on the lower limbs affects the rheological properties of blood (blood count, red blood cell deformability and aggregation, and blood viscosity), lipid profile (total cholesterol, triglycerides, low-density lipoprotein, and high-density lipoprotein), and renal profile (urea, creatinine, and estimated glomerular filtration rate) in young, healthy women. **Methods:** The study group consisted of 15 healthy women aged 20–26 (22.5 ± 1.5), without chronic diseases and not practicing competitive sports. The participants underwent a series of 10 lower limb pressotherapy treatments. A single treatment lasted 30 min and each time the pressure used during the treatment was individually selected according to the participants’ preference. The first blood test was performed a week before the treatments; the second on the day of the start of treatment, but before the pneumatic massage; the third after the completed series of pressotherapy treatments; and the fourth a week after the completed series of treatments. **Results:** In the conducted study, the analysis of the values of the complete blood count showed the following: a significant decrease in red blood cell count, hemoglobin, average hemoglobin concentration in erythrocytes, average red blood cell volume, average hemoglobin mass in red blood cells; a significant increase in average red blood cell volume; and an average hemoglobin mass in red blood cells. The analysis of the values of rheological parameters showed the following: a significant decrease in elongation indices 0.58, 1.13, 4.24, 15.95, 30.94, and 60.00; blood viscosity; the aggregation index; the degree of complete aggregation; and a significant increase in elongation indices 0.30, 1.13, 8.23, 30.94, 60.00; blood viscosity; the degree of complete aggregation; and the half-time of complete aggregation. A decrease in the concentration of low-density lipoprotein and high-density lipoprotein fractions was also noted. No significant changes were found in the values of total cholesterol and triglycerides, as well as in renal profile elements. **Conclusions:** The application of a series of 10 lower limb pressotherapy treatments has a beneficial effect, with a decrease in blood viscosity and the aggregation index, and an increase in the elongation index at shear stress from 0.30 [Pa] to 8.23 [Pa] in young, healthy women. A series of 10 lower limb pressotherapy treatments may affect the decrease in the values at high shear stress forces of 30.95 [Pa] and from 60.00 [Pa] in young, healthy women. The use of a series of 10 lower limb pressotherapy treatments increases the values of hemoglobin, the average red blood cell volume, and the average hemoglobin concentration in erythrocytes, and also reduces the values of red blood cell count, average hemoglobin mass in red blood cells and low-density lipoproteins and high-density lipoproteins in young, healthy women (it also does not cause any adverse changes). The use of pressotherapy on the lower limbs seems to be an effective element of the multi-component prevention of circulatory system diseases.

## 1. Introduction

Pressotherapy includes methods of therapy related to pressure. These include the following: intermittent pneumatic compression (also known as pneumatic massage), compression clothing, and multi-layer bandaging [1]. Pressotherapy is very often used in the process of anti-edema therapy as one of the elements of Comprehensive Anti-Edema Therapy [2] for the prevention of peripheral circulatory failure [3], scar therapy (especially post-burn) [4], or in cosmetology [5]. However, this method is increasingly used to accelerate regeneration in athletes, including after injuries [6,7].

Pressotherapy affects the human body by means of pressure, which causes a local state of hypoxia. Then, blood vessels dilate and blood flow with nutrients through the capillaries increases, as a result of which the procedure accelerates the regenerative processes of soft tissues [8]. Additionally, lymph drainage to proximal parts is facilitated, and it is necessary that in the case of intermittent pneumatic compression, the cuffs are filled with air in the direction of the heart [9]. Importantly, the flow of the circulatory system improves not only quantitatively (increased flow), but also qualitatively (increased flow in a specific direction) [10]. Through applied pressure, the direction of blood and lymph flow is correct, venous reflux is reduced, and thus, venous hypertension is prevented, which, according to Barańska-Rybak and Komorowska (2012), is the primary (among many other risk factors) cause of venous system diseases [11]. As a result of chronic venous insufficiency, leg ulcers often develop, which are a medical, aesthetic, and socio-economic problem [12,13]. They constitute 70–80% of all chronic wounds [14]. Pressotherapy is also used in this case and is considered the gold standard in the treatment and prevention of venous ulcers [14,15]. In the case of venous ulcers, multilayer compression therapy is most often used [16]. Considering the above-mentioned effects on the circulatory system, it can be seen that the use of pressotherapy translates into the regeneration of areas affected by ulcers through better tissue nourishment and the elimination of factors related to lower limb ischemia. It is important that the direction of the pressotherapy is consistent with the direction of venous blood and lymph flow [5,17]. In addition, the pressure should be graduated, i.e., stronger in the distal areas and weaker proximally [3,18,19], which is possible due to the anatomical structure of the limb, which expands proximally. Laplace’s law is applied for this purpose [19], which in this case refers to the dependence of the pressure value on the radius of curvature [20]. More precisely, the pressure in the limb generated (by pressure on this limb) is inversely proportional to the radius of the limb and directly proportional to the tension of the bandage [19].

In cosmetology, the influence of pressotherapy on the circulatory system and lymphatic system is used. The aim of treatments in this field is to improve the functioning of the above-mentioned systems [21], which has a beneficial effect on the metabolism of adipose tissue and reduces the volume of adipocytes [22]; as a result pressotherapy is one of the most effective methods of combating cellulite, i.e., degenerative changes in the subcutaneous tissue on an edematous–fibrotic–sclerotic basis [5]. Additional functions for cosmetic purposes include the removal of excess fluids and toxins from the places of deposition [23]. In cosmetology, there are pressotherapy devices enriched with thermal functions, which significantly accelerate metabolic processes. Preparations applied to the skin are also used, containing active substances, for example with a slimming effect, which are combined with the pneumatic massage procedure [22].

Studies show that the use of intermittent pneumatic compression is also recommended in the case of fractures, as it supports the treatment of bone defects. The cyclic action of positive and negative pressure during pressotherapy stimulates the production of important mediators such as nitric oxide, prostacyclin, and the plasminogen activator. Nitric oxide is a key mediator released by the endothelium, as it causes vasodilation, and thus, leads to a decrease in blood pressure. In addition, it mediates various processes that cause the inhibition of proinflammatory cytokines, which leads to a reduction in the inflammatory response. Nitric oxide also inhibits proliferation, i.e., the multiplication of smooth muscle cells and platelet aggregation, which may consequently lead to the formation of atherosclerotic plaque and thrombi [23,24,25]. A beneficial effect of intermittent pneumatic compression on fibrinolysis processes and a reduction in leukocyte adhesion as well as on the ultrastructural structure of the vein wall and cytokines was also observed [26].

The aim of the study was to investigate whether and how a series of pressotherapy treatments on the lower limbs affects the rheological properties of blood (blood count, red blood cell deformability and aggregation, and blood viscosity), the lipid profile (total cholesterol, triglycerides, and LDL and HDL cholesterol) and the renal profile (urea, creatinine, and eGFR) in young, healthy women.

## 2. Materials and Methods

### 2.1. Participant Characteristics

The study group consisted of 15 healthy women aged 20–26 years (22.5 ± 1.5), with a body height of 158–175 cm (164.6 ± 5.7), a body weight of 49.8–74.4 kg (62.6 ± 8.1), a BMI of 19.5–26.8 kg/m^2^ (23.1 ± 2.4), without chronic diseases, and not practicing competitive sports. The inclusion criteria for the study included the following: aged 20–26 years, female gender, no chronic diseases, and no contraindications to intermittent pneumatic compression (a fever above 38 °C, infectious diseases, neoplasms, acute and subacute inflammation, enlarged lymph nodes, purulent conditions regardless of location, advanced atherosclerosis, any venous diseases of the lower limbs, capillary fragility, skin damage, pain or numbness of an unknown origin, hypertension, asthma, heart failure, renal failure, or pregnancy). The exclusion criteria included the following: competitive sports, lifestyle change during and immediately before the research, diet change during and immediately before the research (the patients’ diet was not monitored), and the consumption of more than 4 cups of coffee per day or more than 2 alcoholic beverages per day.

### 2.2. Measuring Tools

In order to analyze the parameters of blood, blood samples were collected from the participants four times. The first blood test was performed a week before the start of the treatments, the second test was performed on the first day of the treatments (before they started), the third test was performed after the series of 10 treatments had been completed, and the fourth blood test was performed after a week’s break from the third test. The participants underwent 10 pressotherapy treatments from Monday to Friday for two weeks. Blood collection for testing was performed on an empty stomach in the morning between 6:30 and 7:30 a.m. by a qualified nurse. Blood testing was collected from the basilic, cephalic, or median vein of the elbow into test tubes with EDTA K2 (2 × 4.9 mL) (Sarstedt, Nümbrecht, Germany) in accordance with applicable standards.

Blood morphology was tested using an ABX Micros 60 (Horiba Medical, Irvine, CA, USA) device. Complete blood counts were tested as follows: WBC [10^9^/L]—white blood cell count, RBC [10^12^/L]—red blood cell count, HCT [%]—hematocrit, HGB [g/dL]—hemoglobin, MCH [pg]—average hemoglobin mass in red blood cells, MCHC [g/dL]—average hemoglobin concentration in erythrocytes, MCV [fL]—average red blood cell volume, PLT [10^9^/L]—platelet count.

Blood rheological properties were tested using a LORCA analyzer (Laser-Optical Rotational Cell Analyzer, RR Mechatronics, Zwaag, The Netherlands) to examine the aggregation and deformability of red blood cells. The results were presented as the elongation index (EI) and the aggregation index (AI), according to the Hardeman method. Blood analysis was performed within 30 min of collection, at 37 °C, according to standard procedures. In addition, the following measurements were also examined: AMP [au]—degree of complete aggregation, T ½ [s]—half-time of complete aggregation [27,28,29].

For the analysis of blood viscosity, 0.5 mL of plasma was used, which was obtained after centrifugation of morphological blood components. Then, the plasma was placed in a measuring capillary, and the device measured the time in which the plasma passed from point L3 to L4 at a constant pressure and temperature. The normal range is in the range of 1.10 mPa × s–1.90 mPa × s. The study was carried out on a Myrenne Roetgen device (D-52159, Myrenne GMBH, Roetgen, Germany), in order to obtain accurate results, and the device was calibrated using two solutions, NP1 and NP2, respectively, for measurements in the low and high range.

A Cobas C501 (Roche, Porterville, CA, USA) biochemical analyzer was used to examine the lipid profile. The following parameters were determined from blood serum samples: total cholesterol [mmol/L], triglycerides [mmol/L], HDL cholesterol [mmol/L], and LDL cholesterol [mmol/L].

The Cobas C501 (Roche, USA) biochemical analyzer was used to examine the renal profile elements. The following parameters were determined from blood serum samples: urea [mmol/L], creatinine [μmol/L], and eGFR [mL/min/1.73 m^2^].

### 2.3. Description of the Intervention

The study was conducted at the Academy of Physical Education in Krakow over a period of four weeks, during which the participants underwent a series of ten pressotherapy treatments and measurements of physiological parameters. The pressotherapy treatments were conducted daily for 30 min using a CarePump Expert8 (Bardomed LLC, Krakow, Poland) device supporting eight-chamber cuffs for the lower limbs. Each time, the pressure used during the treatment was individually selected to suit the participants’ feelings—maximum tolerated pressure—without causing pain.

Parameters used during the treatment were as follows:

- Gradient: 1 mmHg (a pressure drop in each subsequent chamber by 1 mmHg from the set initial pressure for the first chamber),
- Hold: 3 s (the time the chambers maintain the maximum, set pressure),
- Interval: 3 s (the time between filling each subsequent chamber),
- Output pressure individually selected to suit the participants’ feelings [mmHg] (without pain, maximum tolerated pressure).

### 2.4. Ethics Approval and Informed Consents

The presented prospective, controlled study was consistent with the assumptions of the Helsinki Declaration; approval number of the Bioethical Committee of the District Medical Chamber in Krakow: 64/KBL/OIL/2024 of 4 July 2024; each volunteer read the information about the study design and was given the opportunity to ask questions, after which they gave informed written consent to participate in the study. A physiotherapist took care of the participants’ safety.

### 2.5. Statistical Analysis

Descriptive statistics were determined, including the mean as well as the standard deviation. The normality of distributions was verified with the Shapiro–Wilk test. In order to determine the level of significance of changes within the study group depending on the moment of measurement, the ANOVA/MANOVA test for repeated measurements was used. In the case of statistically significant differences in the analysis of variance, post-hoc tests were performed (Dunn’s multiple comparisons of mean ranks for all tests). A significance level of *p* = 0.05 was assumed in the analyses. The analyses were performed with the use of the Statistica 13 package (Tibco Software Inc., Palo Alto, CA, USA).

## 3. Results

**Figure 1:**
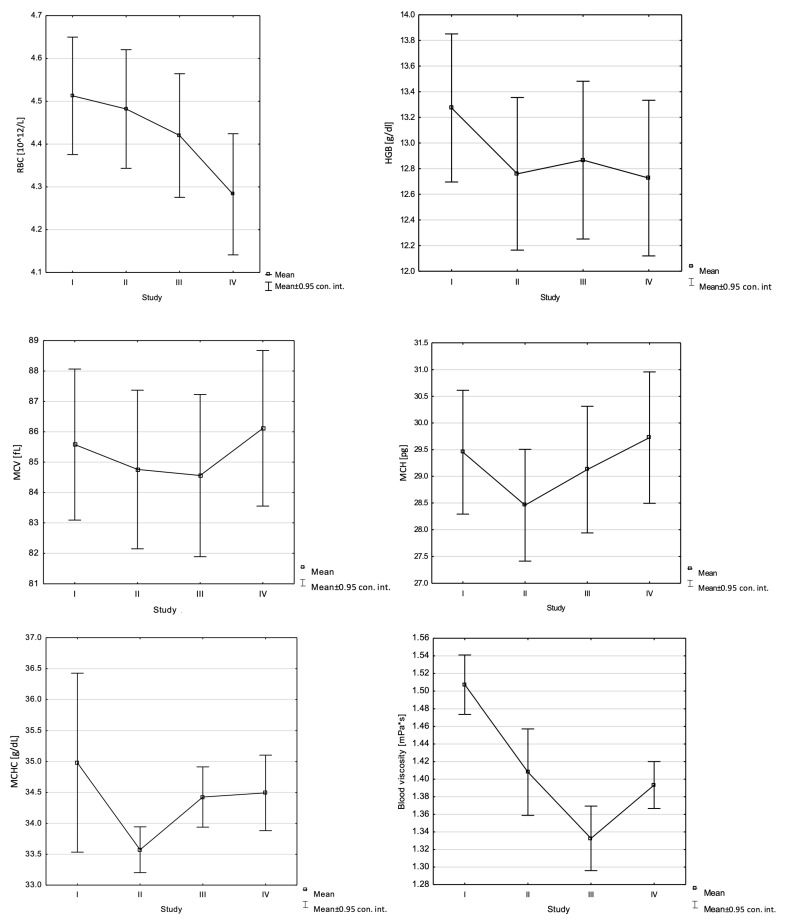

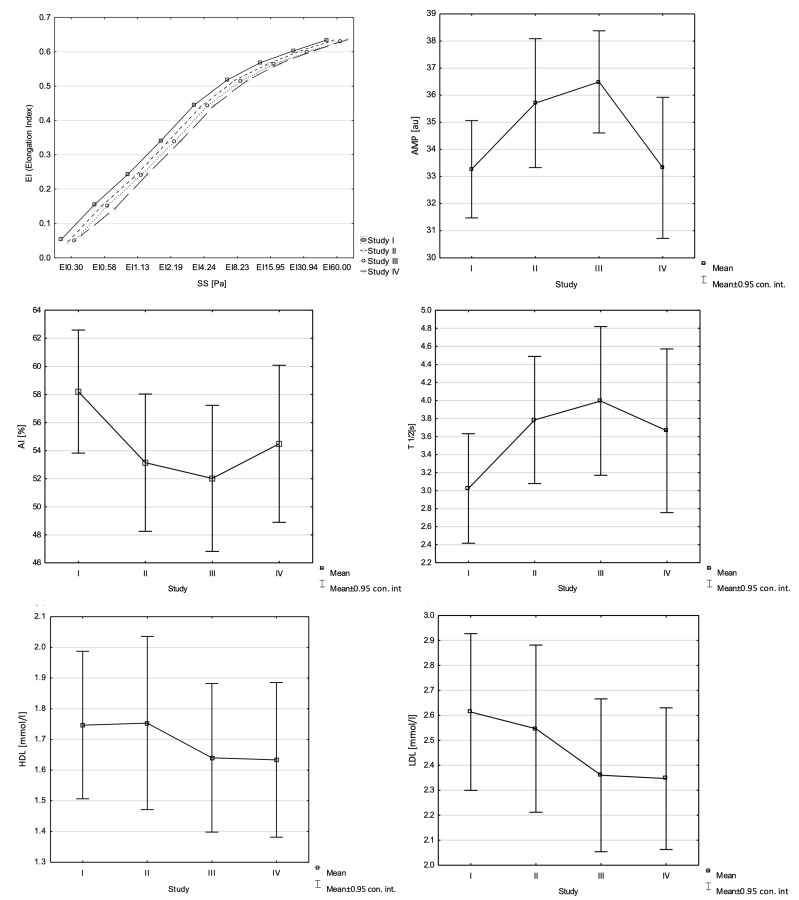
Changes in the studied indicators in the study group (statistically significant).

The analyses included 15 healthy women aged 20–26 years (22.5 ± 1.5) with a BMI of 19.5–26.8 kg/m^2^ (23.1 ± 2.4). Statistical analysis of changes in mean values for the studied parameters showed significant changes between the studies:

- Decrease: RBC (F = 7.96, *p* = 0.00), HGB (F = 6.46, *p* = 0.00), MCHC (F = 3.03, *p* = 0.04), MCV (F = 14.65, *p* = 0.00), MCH (F = 34.22, *p* = 0.00), EI 0.58 (F = 8.87, *p* = 0.00), EI 4.24 (F = 3.36, *p* = 0.03), EI 15.95 (F = 4.76, *p* = 0.01), AI (F = 4.00, *p* = 0.01), blood viscosity (F = 19.84, *p* = 0.00), EI 1.13 (F = 5.31, *p* = 0.00), EI 30.94 (F = 5.50, *p* = 0.00), EI 60.00 (F = 7.5, *p* = 0.00), AMP (F = 6.19, *p* = 0.00), HDL (F = 4.69; *p* = 0.01), LDL (F = 4.08; *p* = 0.01);
- Increase: MCV (F = 14.65, *p* = 0.00), MCH (F = 34.22, *p* = 0.00), EI 0.30 (F = 8.19, *p* = 0.00), EI 8.23 (F = 4.09, *p* = 0.01), T1/2 (F = 4.22, *p* = 0.01), blood viscosity (F = 19.84, *p* = 0.00), EI 1.13 (F = 5.31, *p* = 0.00), EI 30.94 (F = 5.50, *p* = 0.00), EI 60.00 (F = 7.5, *p* = 0.00), AMP (F = 6.19, *p* = 0.00) (Table 1 and Figure 1).

The differences between the individual studies are listed in Table 2.

## 4. Discussion

Pressotherapy significantly increases blood flow, which performs many important functions in the body, including providing transport for oxygen and carbon dioxide between the respiratory system and the tissues, delivering nutrients to the tissues, and also delivering metabolic products [30]. Delis et al. (2000) [31], in their study, examined the effect of intermittent pneumatic compression on the hemodynamics of the popliteal artery. The studies were conducted on healthy people and people with intermittent claudication. The authors of the study showed that the volumetric flow of the popliteal artery increased significantly during the procedure, and decreased after its completion, but was still higher than the initial level. An increase in flow was observed in both groups, but in the group of healthy people, the increase was much greater [31]. Considering the functions that blood performs in the body, it can be concluded that the increase in blood flow is a beneficial phenomenon, as it will accelerate blood functions and support the body in regeneration. Intermittent pneumatic compression also significantly affects the fibrinolytic activity of the vessel walls, which is of great importance in maintaining the appropriate fluidity of the blood [32]. According to the study by Comerot et al. (1997), the use of pneumatic compression once a week for a period of five weeks helps to increase fibrinolytic activity [33]. The changes that were presented in the studies may suggest that intermittent pneumatic compression procedures will also affect changes in blood rheology.

The study conducted in our own work aimed to check what changes in blood rheology would be brought about by the use of a series of 10 pressotherapy treatments for two weeks in young, healthy women. It was suspected that in the conducted studies, a decrease in blood viscosity could occur because pressotherapy causes an increase in its flow velocity [32], and as results from the studies by Libionka et al. (2005) have shown, an increase in blood flow velocity affects the decrease in blood viscosity [34]. In our own study, a decrease in the value of blood viscosity was demonstrated in comparison to the initial study. These results show that under the influence of intermittent pneumatic compression, blood viscosity decreases.

An important factor influencing blood viscosity is the deformability of red blood cells. At high shear forces, the deformability values of erythrocytes are higher, which causes blood viscosity to decrease, which is associated with better flow in microcirculation [35,36]. Shear forces stimulate the production and release of endothelial vasodilators responsible for the expansion of blood vessels, especially nitric oxide. They promote muscle relaxation and increase blood flow to the stimulated or active area [37]. During the analysis of the elongation index results in our study, an increase in the value of this parameter can be observed after the application of a series of treatments at shear stress from 0.30 [Pa] to 8.23 [Pa]; in the study after a break from the treatments, a decrease in the value was observed at a shear stress level of 0.58 [Pa] and from 2.19 [Pa] to 8.23 [Pa]. For a shear stress level of 15.95 [Pa], no change in the parameter value was observed after the applied treatments; however, a decrease was observed after a break from the treatments. The studies also showed a decrease in the value of the tested parameter after the application of pressotherapy at shear stress levels of 30.94 [Pa] and 60.00 [Pa], and an increase in the values of these parameters after a break from the treatments.

Similarly to blood viscosity measurements, the study noted a significant decrease in the AI during the use of intermittent pneumatic compression and an increase in its value after discontinuing the procedures; however, compared to the initial value, a favorable change was noted in the form of a decrease in the AI. Hematocrit is a factor that has a significant effect on blood viscosity and the aggregation index.

Higher hematocrit values cause an increase in the aggregation index, which in turn increases blood viscosity. However, no significant changes were noted in our own study for hematocrit values. No significant changes were shown for WBC and PLT either. The authors’ study showed changes in blood morphology for RBC, HGB, MCV, MCH, and MCHC. Analyzing the changes in RBC values, their decrease can be observed; for HGB and MCHC, there is also a decrease in the values in the tests before and after the end of the procedures, while in the tests performed before and after the series of procedures, an increase in the values for these parameters was observed. A decrease in HGB and MCHC values may indicate anemia [38]; therefore, an increase in these parameters during pressotherapy procedures is a positive phenomenon. In our study, an increase in the MCV value was also observed compared to the first study; however, in the studies before and after the use of pressotherapy, a decrease was observed. For MCH, an increase in the value was observed both during the use of pressotherapy procedures and after a break after their completion.

Diseases account for over 80% of all deaths globally, which is why prevention of these diseases is a very important element. These diseases include, among others, the following: cardiovascular diseases, obesity, type 2 diabetes, hypertension, chronic kidney disease, AIDS, anorexia, and bulimia [39]. Diseases of the circulatory system are the most common cause of death in Poland (almost 50% of all deaths), as presented in research by Migdał (2007) [40]. One of the most important risk factors for cardiovascular diseases is high cholesterol, or more precisely, increased LDL fraction levels with a simultaneous reduction in the HDL fraction and too high a triglyceride concentration, the so-called lipid triad [41]. This study examined the effect of pressotherapy treatments on the level of total cholesterol, HDL and LDL cholesterol fractions, and triglycerides, i.e., parameters that make up the blood lipid profile. This study showed that the level of LDL and HDL fractions changed significantly. Both parameters decreased, which may suggest a beneficial effect on the prevention of cardiovascular diseases and events in the future due to the reduction in LDL levels. The concentration of the LDL fraction decreased by 9.96% and the HDL fraction decreased by 6.86%, which means that the HDL/LDL ratio changed favorably for the health of the circulatory system. It is worth noting that despite the completed series of treatments and a one-week break, a minimal further decrease in LDL and HDL concentrations was noted. The series of treatments lasted 10 working days, which is definitely too short of a time for the prevention of circulatory system diseases. However, research by Russo et al. (2023) shows that the level of LDL is closely related to the incidence of type II diabetes, especially in elderly patients who present a high cardiovascular risk [42]. Approximately 32.2% of all persons with type II diabetes are also affected by cardiovascular disease, which is a common cause of mortality amongst the population [43]. Looking at the long-term effects of pressotherapy, it can be concluded that it reduces the risk of developing type II diabetes, which indirectly reduces the risk of cardiovascular diseases. It seems reasonable to conduct studies using pressotherapy for a longer period and verify its effect on cholesterol levels. It would be necessary to observe whether changes in the concentration of the LDL and HDL fractions occur at the same rate and whether the effect is maintained in the long term. This study did not demonstrate any statistically significant changes in total cholesterol levels, but a downward trend was observed, and continued research in this direction is recommended.

A significant limitation of the conducted study was the small number of subjects, the relatively short time of the study, and the lack of monitoring of diet. In order to obtain more reliable results, it is necessary to extend the studies to a larger number of participants, extend the observation time, and create a separate control or comparative group. In addition, it would be worth conducting studies in different age groups and health states, performing a peripheral blood smear. Although the current studies give promising results regarding the use of pressotherapy, further studies are necessary to fully understand its effect on rheological and biochemical blood indices. Extending these studies may significantly contribute to deepening knowledge in this area and determining whether it will be an effective element of multi-component prevention of global diseases.

## 5. Conclusions

- The application of a series of 10 lower limb pressotherapy treatments has a beneficial effect on a decrease in blood viscosity and the aggregation index, and an increase in the elongation index at shear stress levels from 0.30 [Pa] to 8.23 [Pa] in young, healthy women. A series of 10 lower limb pressotherapy treatments may affect a decrease in values at high shear stress forces of 30.95 [Pa] and from 60.00 [Pa] in young, healthy women.
- The use of a series of 10 pressotherapy treatments of the lower limbs increases the values of HGB, MCVm and MCHC, and also reduces the values of RBC and MCH and the concentration of LDL and HDL cholesterol fractions in young, healthy women.
- The application of a series of 10 lower limb pressotherapy treatments has no effect on renal profile parameters creatinine, urea, and eGFR index in young, healthy women (it also does not cause any adverse changes).
- The use of pressotherapy of the lower limbs seems to be an effective element of multi-component prevention of circulatory system diseases.

## Figures and Tables

**Table 1:** The results of the studied parameters (the mean ± standard deviation) and the analysis of variance for the repeated measurement of the studied indicators—F is the test value and *p* is the significance level.

Parametres	I	II	III	IV	F	*p*	Confidence Interval −95%	Confidence Interval 95%
WBC [10^9^/L]	6.97 ± 0.98	6.83 ± 1.32	6.60 ± 1.20	6.48 ± 1.19	0.84	0.48	5.15	11.37
RBC [10^12^/L]	4.51± 0.25	4.48 ± 0.25	4.42 ± 0.26	4.28 ± 0.26	7.96	0.00	2.84	9.17
HGB [g/dL]	13.27 ± 1.04	12.76 ± 1.07	12.87 ± 1.11	12.73 ± 1.10	6.46	0.00	11.44	17.25
HCT [%]	38.57 ± 2.51	61.51 ± 91.47	37.37 ± 2.99	36.86 ± 2.57	1.04	0.39	32.72	56.30
PLT [10^9^/L]	270.80 ± 67.69	264.67 ± 51.52	261.67 ± 51.97	261.87 ± 62.36	0.67	0.58	246.53	277.60
MCV [fL]	85.58 ± 4.49	84.76 ± 4.71	84.56 ± 4.82	86.11 ± 4.62	14.65	0.00	84.23	86.78
MCH [pg]	29.45 ± 2.10	28.46 ± 1.89	29.13 ± 2.14	29.73 ± 2.22	34.21	0.00	27.95	32.78
MCHC [g/dL]	34.98 ± 2.61	33.57 ± 0.67	34.45 ± 0.88	34.49 ± 1.10	3.03	0.04	33.23	37.68
Blood viscosity [mPa × s]	1.51± 0.06	1.41 ± 0.09	1.33 ± 0.07	1.39 ± 0.05	19.84	0.00	1.29	1.36
EI0.30	0.05 ± 0.01	0.05 ± 0.02	0.05 ± 0.02	0.07 ± 0.02	8.19	0.00	0.03	0.06
EI0.58	10.88 ± 41.53	0.15 ± 0.02	0.15 ± 0.02	0.14 ± 0.02	8.87	0.00	0.13	0.16
EI1.13	0.24 ± 0.02	0.24 ± 0.02	0.24 ± 0.02	0.24 ± 0.02	5.31	0.00	0.22	0.25
EI2.19	0.34 ± 0.02	0.34 ± 0.02	0.34 ± 0.02	0.34 ± 0.02	2.69	0.06	0.32	0.34
EI4.24	0.45 ± 0.01	0.44 ± 0.01	0.44 ± 0.01	0.44 ± 0.02	3.36	0.03	0.43	0.45
EI8.23	0.52 ± 0.01	0.51 ± 0.01	0.51 ± 0.01	0.52 ± 0.01	4.09	0.01	0.50	0.52
EI15.95	0.57 ± 0.01	0.56 ± 0.01	0.56 ± 0.01	0.57 ± 0.01	4.76	0.01	0.55	0.57
EI30.94	0.60 ± 0.01	0.60 ± 0.01	0.60 ± 0.01	0.60 ± 0.01	5.50	0.00	0.59	0.60
EI60.00	0.63 ± 0.01	0.63 ± 0.01	0.63 ± 0.01	0.63 ± 0.01	7.50	0.00	0.62	0.63
AMP [au]	33.27 ± 3.24	35.71 ± 4.30	36.49 ± 3.41	33.32 ± 4.70	6.17	0.00	34.60	38.37
AI [%]	58.21 ± 7.90	53.15 ± 8.84	52.03 ± 9.40	54.49 ± 10.12	4.00	0.01	46.82	57.23
T1/2 [s]	3.02 ± 1.10	3.78 ± 1.27	3.99 ± 1.49	3.66 ± 1.64	4.22	0.01	3.17	4.81
TC [mmol/L]	4.47 ± 0.65	4.45 ± 0.75	4.29 ± 0.77	4.23 ± 0.69	2.40	0.08	3.86	4.71
TG [mmol/L]	1.04 ± 0.44	1.08 ± 0.37	1.15 ± 0.56	1.08 ± 0.47	0.36	0.78	0.84	1.46
HDL [mmol/L]	1.75 ± 0.43	1.75 ± 0.51	1.64 ± 0.44	1.63 ± 0.45	4.69	0.01	1.39	1.88
LDL [mmol/L]	2.61 ± 0.57	2.55 ± 0.60	2.36 ± 0.55	2.35 ± 0.51	4.08	0.01	2.05	2.66
Urea [mmol/L]	4.23 ± 0.89	3.95 ± 0.85	4.29 ± 1.18	4.15 ± 1.00	0.75	0.53	3.63	4.94
Creatinine [μmol/L]	70.45 ± 7.43	69.49 ± 9.55	67.89 ± 8.58	69.58 ± 7.88	1.05	0.38	63.13	72.63
eGFR [mL/min/1.73 m^2^]	80.17 ± 6.31	79.67 ± 8.57	66.60 ± 33.59	81.43 ± 5.32	0.49	0.69	24.89	108.30

**Table 2:** Post-hoc test values for the examined parameters.

Parameters	Study	I	II	III	IV
RBC [10^12^/L]	I		0.55	0.08	0.00
	II	0.55		0.23	0.00
	III	0.08	0.23		0.01
	IV	0.00	0.00	0.01	
HGB [g/dL]	I		0.00	0.01	0.00
	II	0.00		0.45	0.81
	III	0.01	0.45		0.32
	IV	0.00	0.81	0.32	
MCV [fL]	I		0.00	0.00	0.05
	II	0.00		0.46	0.00
	III	0.00	0.46		0.00
	IV	0.05	0.00	0.00	
MCH [pg]	I		0.00	0.02	0.04
	II	0.00		0.00	0.00
	III	0.02	0.00		0.00
	IV	0.04	0.00	0.00	
MCHC [g/dL]	I		0.01	0.25	0.31
	II	0.01		0.08	0.06
	III	0.25	0.08		0.89
	IV	0.31	0.06	0.89	
Blood viscosity [mPa × s]	I		0.00	0.00	0.00
	II	0.00		0.00	0.53
	III	0.00	0.00		0.01
	IV	0.00	0.53	0.01	
EI 0.30	I		0.10	0.34	0.01
	II	0.10		0.49	0.00
	III	0.34	0.49		0.00
	IV	0.01	0.00	0.00	
EI 0.58	I		0.08	0.25	0.00
	II	0.08		0.52	0.00
	III	0.25	0.52		0.00
	IV	0.00	0.00	0.00	
EI 1.13	I		0.00	0.32	0.42
	II	0.00		0.06	0.00
	III	0.32	0.06		0.08
	IV	0.42	0.00	0.08	
EI 4.24	I		0.00	0.12	0.22
	II	0.00		0.13	0.06
	III	0.12	0.13		0.72
	IV	0.22	0.06	0.72	
EI 8.23	I		0.00	0.01	0.16
	II	0.00		0.47	0.07
	III	0.01	0.47		0.27
	IV	0.16	0.07	0.27	
EI 15.95	I		0.00	0.00	0.03
	II	0.00		1.00	0.33
	III	0.00	1.00		0.33
	IV	0.03	0.33	0.33	
EI 30.94	I		0.40	0.00	0.88
	II	0.40		0.01	0.32
	III	0.00	0.01		0.00
	IV	0.88	0.32	0.00	
EI 60.00	I		0.21	0.00	0.96
	II	0.21		0.01	0.19
	III	0.00	0.01		0.00
	IV	0.96	0.19	0.00	
AMP [au]	I		0.01	0.00	0.95
	II	0.01		0.41	0.01
	III	0.00	0.41		0.00
	IV	0.95	0.01	0.00	
AI [%]	I		0.01	0.00	0.06
	II	0.01		0.56	0.48
	III	0.00	0.56		0.20
	IV	0.06	0.48	0.20	
T1/2 [s]	I		0.01	0.00	0.03
	II	0.01		0.47	0.68
	III	0.00	0.47		0.26
	IV	0.03	0.68	0.26	
HDL [mmol/L]	I		0.88	0.02	0.01
	II	0.88		0.01	0.01
	III	0.02	0.01		0.88
	IV	0.01	0.01	0.88	
LDL [mmol/L]	I		0.48	0.01	0.01
	II	0.48		0.05	0.04
	III	0.01	0.05		0.89
	IV	0.01	0.03	0.89	

## Data Availability

All data generated or analyzed during this study are included in this published article.
